# Polypharmacy among anabolic-androgenic steroid users: a descriptive metasynthesis

**DOI:** 10.1186/s13011-015-0006-5

**Published:** 2015-03-15

**Authors:** Dominic Sagoe, Jim McVeigh, Astrid Bjørnebekk, Marie-Stella Essilfie, Cecilie Schou Andreassen, Ståle Pallesen

**Affiliations:** Department of Psychosocial Science, University of Bergen, Christiesgate 12, 5015 Bergen, Norway; Centre for Public Health, Liverpool John Moores University, 15-21 Webster Street, Liverpool, L3 2ET UK; Department of Physical Medicine and Rehabilitation, Unit of Neuropsychology, Oslo University Hospital, Kirkeveien 166, Ullevål, Norway; Departments of Surgery and Paediatrics, La General Hospital, PMB Accra, Ghana; The Competence Centre, Bergen Clinics Foundation, Vestre Torggate 11, 5015 Bergen, Norway

**Keywords:** Anabolic-androgenic steroids, Ergogenic aids, Doping, Human enhancement drugs, IPEDs, Polypharmacy, Stacking, Metasynthesis, Qualitative research

## Abstract

**Background:**

As far as we are aware, no previous systematic review and synthesis of the qualitative/descriptive literature on polypharmacy in anabolic-androgenic steroid(s) (AAS) users has been published.

**Method:**

We systematically reviewed and synthesized qualitative/descriptive literature gathered from searches in electronic databases and by inspecting reference lists of relevant literature to investigate AAS users’ polypharmacy. We adhered to the recommendations of the UK Economic and Social Research Council’s qualitative research synthesis manual and the PRISMA guidelines.

**Results:**

A total of 50 studies published between 1985 and 2014 were included in the analysis. Studies originated from 10 countries although most originated from United States (*n* = 22), followed by Sweden (*n* = 7), England only (*n* = 5), and the United Kingdom (*n* = 4). It was evident that prior to their debut, AAS users often used other licit and illicit substances. The main ancillary/supplementary substances used were alcohol, and cannabis/cannabinoids followed by cocaine, growth hormone, and human chorionic gonadotropin (hCG), amphetamine/meth, clenbuterol, ephedra/ephedrine, insulin, and thyroxine. Other popular substance classes were analgesics/opioids, dietary/nutritional supplements, and diuretics. Our classification of the various substances used by AAS users resulted in 13 main groups. These non-AAS substances were used mainly to enhance the effects of AAS, combat the side effects of AAS, and for recreational or relaxation purposes, as well as sexual enhancement.

**Conclusions:**

Our findings corroborate previous suggestions of associations between AAS use and the use of other licit and illicit substances. Efforts must be intensified to combat the debilitating effects of AAS-associated polypharmacy.

## Introduction

Anabolic-androgenic steroid(s) (AAS) refer to testosterone and its synthetic derivatives mainly used nonmedically for enhancing muscle growth and strength, boosting physical activity or sports performance, and for aesthetic purposes as well as for enhancing psychological well-being [1]. AAS are typically used in phases referred to as ‘cycles’: ‘on cycles’ referring to specific periods when the users administer AAS and ‘off cycles’ referring to an AAS-free phase intended to prevent tolerance towards AAS, lessen the possibility of side effects, and allow recovery of natural hormonal functioning. During ‘on cycles’ users sometimes combine different injectable and oral AAS. This phenomenon is referred to as ‘steroid stacking’ or simply ‘stacking’ [2].

There is also a trend referred to as ‘blast and cruise’ or ‘bridging’ – a continuous ‘on cycle’ whereby many users never go off AAS but alternate between periods of high dose intake during a ‘blast’ phase, and low dose intake during a ‘cruise’ phase. Another way of administering AAS is called ‘blitz-cycles’ , which implies rapidly changing AAS with the aim of preventing tolerance and androgen receptor down-regulation. Moreover, many users complement AAS use or stacking with the use of other substances. In this respect, AAS use has been found to be associated with the use of both licit and illicit substances in systematic reviews of predominantly quantitative literature [3,4].

It has been noted that one of the major drawbacks to successful AAS interventions is public health officials’ failure to recognize AAS users’ extensive pharmacological regimen [2]. A synthesis of the qualitative or descriptive literature on polypharmacy by AAS users is, both from a clinical and research perspective, important in order to increase the understanding of the polypharmacy often associated with AAS use. Such a literature review and synthesis is also valuable in terms of the development and strengthening of AAS use and harm reduction interventions as such investigation will deepen existing knowledge on the various substances used and the specific function they serve, which in some cases deviates significantly from their formal medical indications. Furthermore, results of such investigation would complement evidence emanating from a systematic review of mostly quantitative evidence [3] in the effort to elucidate the phenomenon of polysubstance use by AAS users. However, as far as we are aware, a systematic review and synthesis of the qualitative or descriptive literature on polypharmacy by AAS users has not been published.

Against this backdrop, we conducted the first systematic review and synthesis of the qualitative or descriptive studies presenting data on the use of other licit and illicit substances among AAS users. The research questions guiding the present study were: (a) what substances do AAS users report consuming prior to their AAS debut? (b) what ancillary or supplementary substances do AAS users report using? and (c) what reasons do AAS users assign for using these substances?

## Method

### Search strategy and inclusion criteria

We searched in PsycINFO, PubMed, ISI Web of Science, and Google Scholar for literature. For searches in PubMed and ISI Web of Science, ‘anabolic steroid’ , ‘doping’ , and ‘performance enhancing drug’ , were each combined with ‘interview’ , ‘focus group’ , and ‘qualitative’. These combinations were not practical in PsycINFO and Google Scholar as they produced voluminous redundant hits. Thus, ‘anabolic steroid + doping + performance enhancing drug + interview + focus group + qualitative’ was used in searches in PsycINFO and Google Scholar. From a total of 10,106 hits, 7,720 articles were assessed after the removal of duplicates. We also inspected references of relevant studies and searched in online databases and websites.

This search yielded 15 new articles. Based on titles and abstracts, 106 full-text papers were retrieved for screening after initial evaluation of the 7,735 papers. After screening of the 106 full-text papers, 79 papers were deemed relevant for inclusion. Thus, of the 79 papers scrutinized, 50 studies satisfied the following inclusion criteria: (a) studies used qualitative approaches (interviews, focus groups, or case studies) in data collection, (b) studies delineated or described licit and illicit substances used nonmedically by AAS users, and (c) studies were published in English.

**Figure 1:**
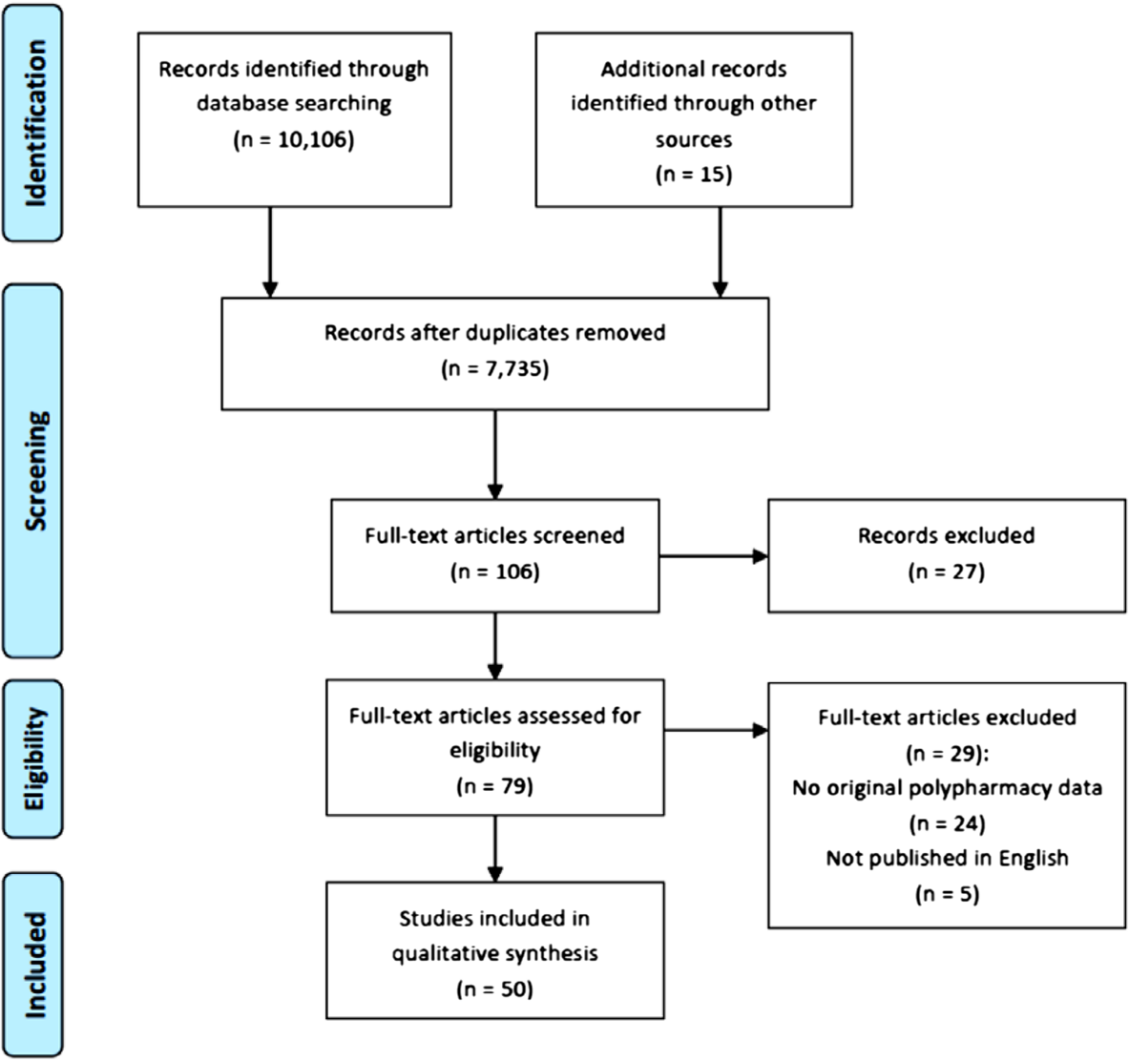
**Flow diagram of systematic literature search.**

We again inspected the characteristics of extracted studies for similarities to curb duplicate extraction and synthesis. The literature search was completed in June 2014. The literature search strategy adhered to Shaw et al.’s [5] recommendations for qualitative literature search as well as the Preferred Reporting Items for Systematic Reviews and Meta-Analyses (PRISMA) guidelines [6]. Figure 1 presents the literature search process.

### Data extraction and synthesis

The first author conducted the study scrutiny and selection. Analysis of the studies was conducted using Smith et al.’s [7] Interpretative Phenomenological Analysis (IPA). Each full-text paper was regarded as a transcript. The first author (DS) read through the full-text papers several times, gaining an overall sense of the themes in the studies through this process. These themes were then highlighted. Using a standardized data extraction form, the first author and another reviewer independently extracted the following data from the included studies: author name and publication year, country, study type, type of AAS users involved in the study, and recruitment site or mode. To assess the quality of the extraction, we calculated inter-reviewer reliability for the two reviewers in SPSS version 20 (IBM Corp.) [8]. DS then independently coded the full-text papers by substance used and reason(s) or motive(s) for use. Study characteristics are presented in Table 1. We have presented all the studies that fall under each substance.

**Table 1:** **Characteristics of qualitative/descriptive studies presenting data on polypharmacy in AAS users**

**First author, year, reference**	**Country**	**Study type**	**AAS users**	**Recruitment site/mode**	**Non-AAS substances ever used**
Ahlgrim 2009 [9]	USA	Case study	41-year-old male former bodybuilder	Hospital	Captopril, carvedilol, digoxin, furosemide, growth hormone, hydrochlorothiazide, spironolactone, torsemide,
Angoorani 2009 [10]	Iran	Interview	843 bodybuilders aged 16 to 40 years	Gymnasium	Amphetamine
Bilard 2011 [11]	France	Interview	203 bodybuilders	Voluntary	Beta-2-agonists, cannabinoids, glucocorticosteroids, peptide hormones
Chandler 2014 [12]	UK	Interview	8 persons	Online forums, syringe exchange center	Aromatase inhibitors, clenbuterol, 2,4-dinitrophenol, clomiphene, diuretics, ephedrine, growth hormone releasing peptide, growth hormone, human chorionic gonadotropin (hCG), insulin-like growth factor 1, insulin, mechano growth factor, melanotan, mephedrone, tamoxifen, thyroid hormones, viagra®/cialis®
Cornford 2014 [13]	England	Interview and focus group	30 males aged 20 to 40 years	Syringe exchange center	Heroin
Davies 2011 [14]	England	Interview and questionnaire^†^	9 male bodybuilders	Gymnasium	Creatine, dietary supplements
Dunn 2010 [15]	Australia	Interview and questionnaire^†^	70 persons	Community	Alcohol, cannabis, cocaine, ecstasy, gamma hydroxybutyrate, hallucinogens, inhalants, ketamine, amphetamine
Filiault 2010 [16]	Australia, Canada, USA	Interview and questionnaire^†^	16 gay male athletes aged 18 to 52 years	Gay sporting groups	Creatine, dietary supplements, growth hormone, recovery drinks
Fudala 2003 [17]	USA	Interview	7 males aged 22 to 33 years	Gymnasium and community	Alcohol, analgesics, cannabis, cocaine, stimulants, growth hormone, human chorionic gonadotropin (hCG), insulin-like growth factor 1
Gårevik 2010 [18]	Sweden	Interview	45 offenders; mean age 30 years	Police station	Amphetamine, anti-oestrogens, benzodiazepines, cannabis, clenbuterol, cocaine, diazepam, ephedra, ephedrine, growth hormone, human chorionic gonadotropin (hCG), heroin, insulin, sildenafil
Goldfield 2009 [19]	Canada	Interview and questionnaire^†^	8 female bodybuilders	Gymnasium	Diuretics, laxatives
Gruber 1998 [20]	USA	Interview	19 female weightlifters	Gymnasium	Clenbuterol, ephedrine, narcotics/other drugs
Gruber 1999 [21]	USA	Interview	5 female bodybuilders	Gymnasium	Alcohol, cannabis, cocaine, clenbuterol, dietary supplements, other drugs, other performance-enhancing drugs
Gruber 2000 [22]	USA	Interview	25 female weightlifters; mean age 31 years	Gymnasium	Aminogluthemide, amphetamine, caffeine, clenbuterol, diuretics, ephedrine, hydroxyl butyrate, human chorionic gonadotropin (hCG), growth hormone, laxatives, nalbuphine, other opioids, tamoxifen, thyroid hormones, yohimbine
Hegazy 2013 [23]	USA	Case study	28-year-old male	Clinic	Alcohol, amphetamine, opioids
Hoff 2012 [24]	Sweden	Interview	11 male (10 powerlifters, 1 weightlifter)	Swedish Sports Confederation	Alcohol, amphetamine, cocaine, narcotics, others
Hope 2013 [25]	England and Wales	Interview and questionnaire^†^	340 injecting drug users	Syringe exchange center	2,4-dinitrophenol, alcohol, amphetamine, anti-oestrogens, clenbuterol, cocaine, ephedrine, erythropoietin, growth hormone, human chorionic gonadotropin (hCG), insulin, melanotan II, nalbuphine, thyroid hormones, diuretics, PDE5i, viagra®/cialis®
Kanayama 2003 [26]	USA	Interview and questionnaire^†^	48 male weightlifters; mean age 29 years	Gymnasium and sports supplement store	Alcohol, cannabis, cocaine, opioids, other narcotics/illicit drugs
Kanayama 2003 [27]	USA	Interview	24 male drug users; mean age 32 years	Clinic	Alcohol, cocaine, heroin, nalbuphine, opioids, oxycodone
Kanayama 2009 [28]	USA	Interview	62 male weightlifters	Gymnasium and sports supplement store	Alcohol, cannabis, cocaine, opioids, other performance-enhancing drugs, other drugs
Katz 1990 [29]	USA	Case study	23-year-old male bodybuilder	Gymnasium	Alcohol, cocaine
Kimergård 2014 [30]	England and Wales	Interview	24 males aged 21 to 61 years; mean age 34 years	Gymnasium, prison, steroid clinic and charity, syringe exchange centre	Amphetamine, clenbuterol, growth hormone, ephedrine, human chorionic gonadotropin (hCG), insulin, melanotan II, sildenafil, tamoxifen
Klötz 2010 [31]	Sweden	Interview	33 male prisoners aged 21 to 52 years	Prison	Antidepressants, anti-oestrogen, aspirin®, benzodiazepines, caffeine, cannabis, central stimulating drugs, clenbuterol, creatine, diuretics, ephedrine, Gamma hydroxybutyrate, insulin-like growth factor 1, insulin, genotropine, muscle relaxing drugs, myoblast, opiates, other drugs, potency increasing drugs, somatotropine, testicular function recovering hormones
Korkia 1993 [32]	England, Scotland, and Wales	Interview	110 persons (13 female) aged 16 to 63 years	Clinic, gymnasium, syringe exchange centre	Antibiotics, corticosteroids, dietary supplements, diuretics, esiclene, human chorionic gonadotropin (hCG), oestrogen-antagonist drug, growth hormone, thiomucase, thyroxine
Korkia 1996 [33]	England	Interview and questionnaire^†^	15 females; mean age 28 years	Not specified	Clenbuterol, growth hormone, nolvadex, nubain®, thiomucase, triacana
Kusserow 1990 [34]	USA	Interview	72 (6 female) persons (mostly adolescents); 14 to 25 years; mean age 20 years	Not specified	Alcohol, blood pressure regulators, ‘downers’, estrogen inhibitors, growth hormone, cannabis, Recreational substances/drugs, ‘uppers’
Larance 2008 [35]	Australia	Interview	60 males aged 17 to 59 years	Gymnasium, internet forums, supplement shops	Anti-oestrogenic agents, aspirin®, benzodiazepines, caffeine, cannabis, cocaine, clenbuterol dehydroepiandrosterone (DHEA), diuretics, ecstasy, hallucinogens, heroin, human chorionic gonadotrophin (hCG), ephedrine, growth hormone, inhalants, insulin-like growth factors, insulin, meth/amphetamine, thyroxine
Lenehan 1996 [36]	England	Interview	386 persons aged 17 to 56 years; mean age 28 years	Gymnasium	Clenbuterol, corticosteroids, diuretics, growth hormone, human chorionic gonadotropin (hCG), thyroxine, insulin-like growth factor 1, nubain®, tamoxifen
Lundholm 2010 [37]	Sweden	Interview	924 (20 female) persons	Prison	Benzodiazepines, cannabis, cocaine, meth/amphetamine, opiates
Malone 1995 [38]	USA	Interview	77 (6 female) powerlifters and bodybuilders	Gymnasium	Alcohol, cocaine, hallucinogen, opioids, sedatives, stimulants, tetrahydrocannabinol, tobacco
McBride 1996 [39]	Wales	Case study	3 males: 1 AAS dealer and roofer aged 27 years, 1 bodybuilder aged 22 years, and 1 gym owner aged 26 years)	Not specified	Amphetamine, cannabis, clenbuterol, human chorionic gonadotropin (hCG), nalbuphine, tamoxifen, temazepam
McKillop 1987 [40]	Scotland	Interview	8 male bodybuilders aged 17 to 32 years	Gymnasium	Furosemide, thiazides, thyroxine, human chorionic gonadotropin (hCG)
Moss 1992 [41]	USA	Interview	50 male bodybuilders	Gymnasium	Clomiphene citrate, human chorionic gonadotropin (hCG)
Moss 1993 [42]	USA	Interview	30 male bodybuilders	Gymnasium	Clomiphene citrate, human chorionic gonadotropin (hCG)
Pappa 2012 [43]	Europe	Interview	9 athletes aged 19 to 26 years	Community via snowball sampling	Analgesics, amphetamine, caffeine, cannabis, dietary supplements, diuretics, erythropoietin.
Perry 1990 [44]	USA	Interview and questionnaire^†^	20 male weightlifters aged 18 to 28 years	Gymnasium	Human chorionic gonadotropin (hCG)
Perry 2003 [45]	USA	Interview	10 male weightlifters aged 21 to 40 years	Gymnasium	Aspirin®, caffeine, clomiphene, creatine, dietary supplement, ephedrine, glutamine, liothyronine, protein powder, yohimbine
Peters 1997 [46]	Australia	Interview and questionnaire^†^	100 persons (6 female) aged 18 to 50 years	Advertisements, gymnasium, interviews, radio, sports shops and associations, syringe exchange centre	Alcohol, aminogluthimide, amphetamine, antibiotics, beta blockers, caffeine, cannabis, chromium picolinate, clenbuterol, cocaine, daonil®, dietary supplement, diuretics, ecstasy, ephedrine, growth hormone, human chorionic gonadotropin (hCG), hydroxocobal amin, insulin-like growth factor 1, insulin, oestrogen antagonist, pregnyl®, proviron®, teroxin (T3), thyroxine
Pope 1988 [47]	USA	Interview	41 male bodybuilders and footballers	Gymnasium	Alcohol, cannabis, cigarettes, cocaine, human chorionic gonadotropin (hCG)
Pope 1994 [48]	USA	Interview	88 athletes; mean age 26 years	Gymnasium	Alcohol, cannabis, tobacco
Rashid 2000 [49]	USA	Case study	40-year-old male	Clinic	Cocaine, cannabis, ‘uppers’, ‘downers’, lysergic acid diethylamide (LSD)
Schäfer 2011 [50]	Denmark	Case study	26-year-old male bodybuilder	Clinic	Erythropoietin
Silvester 1995 [51]	USA	Interview	22 former athletes aged 36 to 66 years	Not specified	Growth hormone
Skårberg 2007 [52]	Sweden	Interview and questionnaire^†^	18 male drug users; mean age 35 years	Clinic	Alcohol, narcotics/other drugs
Skårberg 2008 [53]	Sweden	Interview	6 drug users (2 female)	Clinic	Alcohol, amphetamine, analgesics, anti-catabolics, anti-oestrogens, aspirin®, benzodiazepines, bronchodilators, buprenorphine, caffeine, cannabis, cocaine, codeine, conjugated linoleic acid, creatine, dietary supplements, ecstasy, ephedra, ephedrine, growth hormone, Herbal products, insulin growth factor 1, insulin, protein powder, testosterone releasers
Skårberg 2009 [54]	Sweden	Interview and questionnaire^†^	32 male drug users	Clinic	Alcohol, amphetamine, anti-oestrogen (clomid), analgesics, anti-acne drug, anti-catabolics, anti-depressants, anti-hypertensive drugs, anti-oestrogens, benzodiazepines, bronchodilators, cannabis, cocaine, creatine, dietary supplements, diuretics, ephedrine, fat-loss agents, gamma hydroxybutyrate, growth hormone, heroin, insulin, insulin-like growth factor 1, levodopa, muscle oil (synthol), non-steroidal anti-inflammatory drugs, opioid, plant steroid compounds, protein powder, stimulants, testosterone boosters, thyroid hormone
Strauss 1985 [55]	USA	Interview	10 weight-trained female athletes; mean age 33 years	Personal contact	Acetaminophen, aspirin®, benoxaprofen, Ben-Gay®, caffeine, calcium, choline and inositol, dietary supplements, dimethyl sulfoxide, codeine, electrolyte solution, epinephrine, furosemide, growth hormone, levodopa, lidocaine, naproxen, oxycodone hydrochloride, phenylbutazone, piroxicam, potassium, suntan pills, thyroglobulin, vitamins
Tallon 2007 [56]	Scotland	Interview and questionnaire^†^	30 males aged 18 to 43 years; mean age 27 years	Gymnasium	Alcohol, cannabis, cocaine, clenbuterol, dietary supplements, diuretics, ecstasy, growth hormone, insulin, tamoxifen
Wilson-Fearon 1999 [57]	England	Case study	29-year-old bodybuilder	Not specified	Clenbuterol, dietary supplements, diuretics, growth hormone, human chorionic gonadotropin (hCG), thiomucase
Wines 1999 [58]	USA	Interview	11 weightlifters (5 female) aged 19 to 42 years	Gymnasium	Alcohol, buprenorphine, heroin, hydrocodone, nalbuphine, other drugs

^†^We relied on the qualitative results generated from the interview.

### Classification of substances

We sought to classify the various non-AAS substances used by AAS users into meaningful groups. First, SP provided a functional categorization of the substances. Acknowledging that some AAS users self-administer these substances for purposes contrary to their conventional use, DS built on SP’s classification by allocating the substances into SP’s groups based on motives for use as presented by users in the literature. For substances for which motive for use was not delineated in the literature, DS grouped them based on Evans-Brown et al.’s [2] classification of human enhancement substances and a classification by the Norwegian Institute of Public Health [59]. JM inspected the grouping and provided further advice. Next, DS allocated substances that at this stage could not be allocated into groups based on the three previous methods by referring to Medscape Drug Reference and Wikipedia [60]. We reached consensus on the classification through further review and discussion.

## Results and discussion

### Description of studies and inter-reviewer reliability

A total of 50 studies were included in the metasynthesis. Participants’ ages ranged from 14 [34] to 66 years [51]. The year of publication of the studies ranged from 1985 [55] to 2014 [12,13,30]. Studies originated from 10 countries with the highest number from the United States (*n* = 22), followed by Sweden (*n* = 7), England only (*n* = 5), the United Kingdom (*n* = 4), Australia (*n* = 3), and Scotland only (*n* = 2). Additionally, one study originated from Canada, Denmark, France, Iran, and Wales only respectively. One study [16] originated from Australia, Canada, and USA while another described the sample as European [43]. Thirty studies used interviews [10-12,17,19-22,24,27,28,30-32,34-38,40-43,45,47,48,51,53,55,58], seven were case studies [9,23,29,39,49,50,57], one used interviews and focus groups [13], and twelve [14-16,19,25,26,33,44,46,52,54,56] used interviews supported by a questionnaire. For the studies that used both interviews and questionnaires, we relied on the qualitative or descriptive results generated from the interviews. There was very good agreement (Kappa = 0.82, *p* < 0.001) between the two reviewers [61]. Through further analysis and dialogue agreement was reached on discrepant extractions.

### Substances used prior to AAS initiation

Before their AAS use debut, some users had experimented with or were regular users of other substances. This was presented by ten studies [17,21,24,26-29,34,47,53]. The most prominent of these substances were alcohol, amphetamine, cannabis, and cocaine. Others were analgesics/opioids, heroin, stimulants, and dietary/nutritional supplements such as creatine, and protein powder as well as other unspecified licit and illicit substances (see Table 2).

**Table 2:** **Non-AAS substances used before AAS use debut, reason(s)/motive(s) for use, and studies**

**Substance**	**Reason(s) for use**	**Studies (First author, reference)**
Alcohol	Better sleep and relaxation	Fudala [17]; Gruber [21]; Hoff [24]; Kanayama [26]; Katz [29]; Kusserow [34]; Perry [47]; Skårberg [53]
Amphetamine	Boosting training, alertness, psychological wellbeing	Hoff [24]; Skårberg [53]
Analgesics/opioids^†^	Pain relief	Kanayama [26]
Cannabis	NS	Fudala [17]; Hoff [24]; Kanayama [26]; Kusserow [34]; Perry [47]
Cocaine	NS	Fudala [17]; Gruber [21]; Kanayama [26]; Katz [29]; Perry [47]
Creatine	Boosting training	Skårberg [53]
Dietary/nutritional supplements^†^	Boosting training, energy	Skårberg [53]
Heroin	NS	Cornford [13]
Protein powder	Boosting training	Skårberg [53]
Stimulants^†^	NS	Kusserow [34]
Other IPEDs, licit and illicit substances	NS	Gruber [21]; Hoff [24]; Kanayama [27,28]; Perry [47]

IPEDs: Image and performance-enhancing drugs.

NS: Not specified.

^†^Substance class - not specified

In Kanayama et al.’s study [26]:

[AAS] users displayed much higher rates of other illicit drug use, abuse, or dependence than non-users, with use of other illicit substances almost always preceding first use of AAS (p. 77).

A recent study by Cornford, Kean, and Nash [13] also highlights heroin use as a precursor to AAS use:

A quick way to make yourself look healthy, isn’t it, without being embarrassed about being on heroin [is to use AAS], do you know what I mean. It [heroin] does take a lot of your confidence away don’t it and like I say, especially, I lose weight pretty fast when I’m on heroin, do you know what I mean. It [AAS use] is a quick way to just make yourself look healthy again, isn’t it (p. 2).

Furthermore, it is important to note that our data also suggested that AAS use may precede the use of other substances for some individuals. In Hoff’s study [24]:

Respondent 8 reported using narcotics after he had started using AAS. In this case, alcohol and drug abuse cannot explain why he started doping [using AAS]. However, AAS use seems to have led him into drug use and criminality in order to finance his extensive AAS use and investment in elite powerlifting. This respondent became aggressive and violent when he combined AAS and alcohol. Due to these side effects he changed from alcohol to cocaine as his primary social drug when he was on AAS (p. 63).

### Use of supplementary/ancillary substances

AAS users often engaged in stacking and the use of various licit and illicit substances during their ‘on cycles’ as previously shown. For instance, in a study by McBride [39], “…Mr B had initially used nalbuphine in conjunction with anabolic steroids, clenbuterol, ephedrine, and tamoxifen, all to aid bodybuilding” (p. 69). Indeed, in a study [46] of 100 AAS users: “A number of other drugs were used in addition to AAS as part of their training routine by 49% of the sample” (p. 49).

The most popular supplementary/ancillary substances declared by AAS users in multiple studies were: alcohol, amphetamine/meth, aspirin®, caffeine, cannabis/cannabinoids, clenbuterol, clomiphene citrate, cocaine, codeine, creatine, ephedra/ephedrine, erythropoietin, furosemide, gamma hydroxybutyrate (GHB), growth hormone, heroin, human chorionic gonadotropin (hCG), insulin, insulin-like growth factor 1 (IGF-1), melanotan, nalbuphine/nubain®, protein powder, tamoxifen, thyroxine, and tobacco. Other popular classes of substances presented were analgesics/opioids, anti-oestrogens, benzodiazepines, dietary/nutritional supplements, diuretics, hallucinogens, and stimulants (see Table 3).

**Table 3:** **Use of non-AAS substances, reason(s)/motive(s) for use, and studies**

	**Current polypharmacy (Combined with AAS)**		**Lifetime polypharmacy (Ever use)**		
**Substance**	**Reason(s) for use**	**Studies (First author, reference)**	**Reason(s) for use**	**Studies (First author, reference)**	**Number of studies**
2,4-dinitrophenol	NS	Chandler [12]	NS	Chandler [12]; Dunn [15]; Hope [25]; Larance [35]	4
Acetaminophen	NS	Strauss [55]	NS	Strauss [55]	1
Alcohol	Better sleep and relaxation	Chandler [12]; Hegazy [23]; Kanayama [28]; Kusserow [34]; Lundholm [37]; Malone [38]; Peters [46]; Perry [48]; Skårberg [52-54]	Better sleep and relaxation	Chandler [12]; Dunn [15]; Fudala [17]; Gruber [21,22]; Hegazy [23]; Hoff [24]; Hope [25]; Kanayama [26-28]; Katz [29]; Kusserow [34]; Malone [38]; Peters [46]; Perry [48]; Skårberg [52-54]; Tallon [56]; Wines [58]	23
Aminogluthimide	Reducing receptors’ attraction to cortisol	Peters [46]	Reducing receptors’ attraction to cortisol	Gruber [22]; Peters [46]	2
Amyl nitrate	NS	Chandler [12]	NS	Chandler [12]	1
Analgesics/opioids^†^	Pain relief	Ahlgrim [9]; Hegazy [23]; Kanayama [28]; Klötz [31]; Kusserow [34]; Lundholm [37]; Malone [38]; McBride [39]; Pappa [43]; Skårberg [53]	Pain relief	Ahlgrim [9]; Fudala [17]; Gruber [22]; Hegazy [23]; Kanayama [26,28], Klötz [31]; Kusserow [34]; Malone [38]; McBride [39]; Pappa [43]; Rashid [49]; Skårberg [53,54]	14
Anti-acne drugs^†^	–	–	Combating acne	Skårberg [54]	1
Antibiotics^†^	Combating acne	Peters [46]	Combating acne	Korkia [32]; Peters [46]	2
Anti-catabolics^†^	NS	Skårberg [53]	Facilitating synthesis of hepatic protein and nitrogen economy	Skårberg [53,54]	2
Anti-depressants^†^	Combating side effects	Klötz [31]	Combating side effects, depression relief, boosting levels of serotonin and noradrenaline	Klötz [31]; Skårberg [54]	2
Anti-oestrogens^†^	Burning fat, combating gynecomastia, reducing effects on oestrogen	Klötz [31]; Kusserow [34]; Peters [46]; Skårberg [53]	Combating gynecomastia, burning fat, reducing effects on oestrogen	Gårevik [18]; Hope [25]; Klötz [31]; Korkia [32]; Kusserow [34]; Larance [35]; Peters [46]; Skårberg [53,54]	9
Aromatase inhibitors^†^	NS	Chandler [12]	NS	Chandler [12]	1
Aspirin®	NS	Klötz [31]; Perry [45]; Skårberg [53]; Strauss [55]	NS	Klötz [31]; Larance [35]; Perry [45]; Skårberg [53]; Strauss [55]	5
Ben-Gay®	NS	Strauss [55]	NS	Strauss [55]	1
Benoxaprofen	NS	Strauss [55]	NS	Strauss [55]	1
Benzodiazepines^†^	Better sleep, combating side effects, relaxation	Klötz [31]; Larance [35]; Lundholm [37]; McBride [39]; Skårberg [53]	Combating side effects, enhancing sleep and relaxation, self-control, sedation	Gårevik [18]; Klötz [31]; Larance [35]; Lundholm [37]; Malone [38]; McBride [39]; Skårberg [53,54]	8
Beta blockers^†^	Burning fat	Peters [46]	Burning fat	Peters [46]	1
Beta-2-agonists^†^	–	–	NS	Bilard [11]	1
Blood pressure regulators^†^	NS	Kusserow [34]	Lower blood pressure	Kusserow [34]; Skårberg [54]	2
Bronchodilators^†^	Energy and boosting training	Skårberg [53]	Burning fat, energy and boosting training, increasing strength	Skårberg [53,54]	2
Buprenorphine	NS	Skårberg [53]	NS	Skårberg [53]; Wines [58]	2
Caffeine	Burning fat	Klötz [31]; Pappa [43]; Perry [45]; Peters [46]; Skårberg [53]; Strauss [55]	Burning fat	Gruber [22]; Klötz [31]; Larance [35]; Pappa [43]; Peters [46]; Perry [45]; Skårberg [53]; Strauss [55]	8
Calcium	–	–	NS	Strauss [55]	1
Cannabis/cannabinoids	Enhancing sleep, relaxation	Chandler [12]; Kanayama [27,28]; Klötz [31]; Kusserow [34]; Lundholm [37]; Malone [38]; McBride [39]; Pappa [43]; Peters [46]; Perry [48]; Skårberg [53,54]	Enhancing sleep, relaxation	Bilard [11]; Chandler [12]; Dunn [15]; Fudala [17]; Gruber [21,22]; Hoff [24]; Kanayama [27,28]; Klötz [31]; Kusserow [34]; Larance [35]; Malone [38]; McBride [39]; Pappa [43]; Peters [46]; Perry [48]; Rashid [49]; Skårberg [53,54]; Tallon [56]	21
Captopril	NS	Ahlgrim [9]	NS	Ahlgrim [9]	1
Carvedilol	NS	Ahlgrim [9]	NS	Ahlgrim [9]	1
Choline and inositol	NS	Strauss [55]	NS	Strauss [55]	1
Chromium picolinate	Reducing body weight	Peters [46]	Reducing body weight	Peters [46]	1
Clenbuterol	Anabolic effects, burning fat, removing skin fluid, weight loss	Chandler [12]; Gruber [21]; Kimergård [30]; Klötz [31]; Lenehan [36]; McBride [39]; Peters [46]; Wilson-Fearon [57]	Anabolic effects, burning fat, removing skin fluid, weight loss	Chandler [12]; Gruber [20-22]; Hope [25]; Kimergård [30]; Klötz [31]; Korkia [33]; Larance [35]; Lenehan [36]; McBride [39]; Peters [46]; Tallon [56]; Wilson-Fearon [57]	14
Clomiphene citrate	NS	Chandler [12]; Moss [41,42]; Perry [45]	NS	Chandler [12]; Moss [41,42]; Perry [45]	4
Cocaine	Boosting training, burning fat, increasing strength	Chandler [12]; Kanayama [28]; Larance [35]; Lundholm [37]; Malone [38]; Peters [46]; Skårberg [53,54]	Boosting training, burning fat, increasing strength	Chandler [12]; Dunn [15]; Fudala [17]; Gårevik [18]; Gruber [21]; Hoff [24]; Hope [25]; Kanayama [26-28]; Katz [29]; Larance [35]; Lundholm [37]; Malone [38]; Peters [46]; Rashid [49]; Skårberg [53,54]; Tallon [56]	19
Codeine	Boosting training	Skårberg [53]; Strauss [55]	Boosting training	Skårberg [53]; Strauss [55]	2
Conjugated linoleic acid	Burning fat	Skårberg [53]	Burning fat	Skårberg [53]	1
Corticosteroids^†^	NS	Lenehan [36]	NS	Bilard [11]; Korkia [32]; Lenehan [36]	3
Creatine	Enhancing the effects of training	Klötz [31]; Skårberg [53]; Perry [45]	Anabolic effects, endurance booster, enhancing the effects of training, recovery aid	Davies [14]; Filiault [16]; Hoff [24]; Klötz [31]; Perry [45]; Skårberg [53,54]	7
Daonil®	Increasing insulin release	Peters [46]	Increasing insulin release	Peters [46]	1
Dehydroepiandrosterone (DHEA)	–	–	NS	Larance [35]	1
Diazepam	–	–	NS	Gårevik [18]	1
Dietary/nutritional supplements^†^	Energy and boosting training, nutrition	Pappa [43]; Perry [45]; Peters [46]; Skårberg [53,54]; Strauss [55]; Wilson-Fearon [57]	Energy and boosting training, anabolic effects, endurance booster, nutrition, recovery aid	Davies [14]; Filiault [16]; Gruber [21]; Korkia [32]; Pappa [43]; Perry [45]; Peters [46]; Skårberg [53,54]; Strauss [55]; Tallon [56]; Wilson-Fearon [57]	12
Digoxin	NS	Ahlgrim [9]	NS	Ahlgrim [9]	1
Dimethyl sulfoxide	NS	Strauss [55]	NS	Strauss [55]	1
Diuretics^†^	Combating side effects, masking doping drugs, reducing fluid levels	Chandler [12]; Klötz [31]; Lenehan [36]; Pappa [43]; Peters [46]; Wilson-Fearon [57]	Combating side effects, masking doping drugs, reducing fluid levels	Chandler [12]; Goldfield [19]; Hope [25]; Klötz [31]; Korkia [32]; Larance [35]; Lenehan [36]; Pappa [43]; Peters [46]; Skårberg [54]; Tallon [56]; Wilson-Fearon [57]	12
Electrolyte solution	NS	Strauss [55]	NS	Strauss [55]	1
Ephedra/Ephedrine	Energy and boosting training, enhancing weight loss	Chandler [12]; Kimergård [30]; Klötz [31]; McBride [39]; Perry [45]; Peters [46]; Skårberg [53]	Energy and boosting training, burning fat, enhancing weight loss, increasing strength	Chandler [12]; Gårevik [18]; Gruber [20]; Gårevik [18]; Hope [25]; Kimergård [30]; Klötz [31]; Larance [35]; McBride [39]; Perry [45]; Peters [46]; Skårberg [53,54]	14
Epinephrine	NS	Strauss [55]	NS	Strauss [55]	1
Erythropoietin (EPO)	NS	Pappa [43]; Schäfer [50]	NS	Hope [25]; Pappa [43]; Schäfer [50]	3
Esiclene	–	–	NS	Korkia [32]	1
Fat-loss agents^†^	–	–	Burning fat	Skårberg [54]	1
Furosemide	Weight loss	Ahlgrim [9]; Strauss [55]	Weight loss	Ahlgrim [9]; McKillop [40]; Strauss [55]	3
Gamma hydroxybutyrate (GHB)	Enhancing sleep	Chandler [12]; Klötz [31]; Skårberg [54]	Enhancing sleep	Chandler [12]; Dunn [15]; Gruber [22]; Klötz [31]; Skårberg [54]	5
Genotropine	NS	Klötz [31]	NS	Klötz [31]	1
Glutamine	NS	Perry [45]	NS	Perry [45]	1
Growth hormone	Anabolic effects and strength, burning fat, weight loss	Chandler [12]; Kimergård [30]; Kusserow [34]; Lenehan [36]; Peters [46]; Skårberg [53,54]; Strauss [55]; Wilson-Fearon [57]	Anabolic effects, burning fat, endurance booster, recovery aid, weight loss	Chandler [12]; Filiault [16]; Fudala [17]; Gårevik [18]; Hope [25]; Kimergård [30]; Korkia [32,33]; Kusserow [34]; Larance [35]; Lenehan [36]; Peters [46]; Silvester [51]; Skårberg [53,54]; Strauss [55]; Tallon [56]; Wilson-Fearon [57]	18
Growth hormone releasing peptides^†^	NS	Chandler [12]	NS	Chandler [12]	1
Hallucinogens^†^	NS	Larance [35]; Malone [38]	NS	Dunn [15]; Larance [35]; Malone [38]	3
Herbal products^†^	NS	Skårberg [53]	Increasing strength	Skårberg [53,54]	
Heroin	Enhancing sleep, pain relief	Cornford [13]; Larance [35]; Skårberg [54]	Enhancing sleep, pain relief	Gårevik [18]; Cornford [13]; Kanayama [27]; Larance [35]; Skårberg [54]; Wines [58]	6
Human chorionic gonadotropin (hCG)	Minimizing depressive symptoms upon AAS cessation/withdrawal, improving testosterone production, preventing weight loss, stopping testicular atrophy, increasing strength	Chandler [12]; Kimergård [30]; Lenehan [36]; McBride [39]; Moss [41,42]; Perry [44]; Peters [46]; Perry [47]; Korkia [32]; Wilson-Fearon [57]	Anabolic effects, increasing testosterone production, minimizing depressive symptoms upon AAS cessation/withdrawal, preventing weight loss, stopping testicular atrophy, increasing strength	Chandler [12]; Fudala [17]; Gruber [22]; Gårevik [18]; Hope [25]; Kimergård [30]; Korkia [32]; Larance [35]; Lenehan [36]; McBride [39]; McKillop [40]; Moss [41,42]; Perry [44]; Peters [46]; Perry [47]; Wilson-Fearon [57]	17
Hydrochlorothiazide	Weight loss	Ahlgrim [9]	Weight loss	Ahlgrim [62]	1
Hydrocodone	–	–	NS	Wines [58]	1
Hydroxocobal amin	Weight gain	Peters [46]	Weight gain	Peters [46]	1
Inhalants^†^	NS	Larance [35]	NS	Dunn [15]; Larance [35]	2
Insulin	Anabolic effects and strength, burning fat, weight loss	Chandler [12]; Kimergård [30]; Klötz [31]; Peters [46]; Skårberg [53,54]	Anabolic effects and strength, burning fat, weight loss	Chandler [12]; Gårevik [18]; Hope [25]; Kimergård [30]; Klötz [31]; Larance [35]; Peters [46]; Skårberg [53,54]; Tallon [56]	10
Insulin-like growth factor 1 (IGF-1)	Anabolic effects and strength, burning fat	Chandler [12]; Klötz [31]; Lenehan [36]; Peters [46]; Skårberg [53,54]	Anabolic effects and strength, burning fat	Chandler [12]; Fudala [17]; Klötz [31]; Larance [35]; Lenehan [36]; Peters [46]; Skårberg [53,54]	8
Ketamine	NS	Chandler [12]	NS	Chandler [12]; Dunn [15]	2
Laxative	–	–	NS	Goldfield [19]	2
Levodopa	NS	Strauss [55]	Increasing growth hormone	Strauss [55]; Skårberg [54]	2
Lidocaine	NS	Strauss [55]	NS	Strauss [55]	2
Liothyronine	NS	Perry [45]	NS	Perry [45]	1
Lysergic acid diethylamide (LSD)	NS	Skårberg [54]	NS	Rashid [49]; Skårberg [54]	2
Mechano growth factor	NS	Chandler [12]	NS	Chandler [12]	1
Melanotan	Boosting training, skin tanning	Chandler [12]; Kimergård [30]	Boosting training, skin tanning	Chandler [12]; Hope [25]; Kimergård [30]	3
Mephedrone	NS	Chandler [12]	NS	Chandler [12]	1
Meth/amphetamine	Alertness, boosting training, burning fat, increasing aggression during exercise, increasing strength, psychological wellbeing	Chandler [12]; Hegazy [23]; Kimergård [30]; Larance [35]; Lundholm [37]; McBride [39]; Pappa [43]; Peters [46]; Skårberg [53,54]	Alertness, boosting training, burning fat, increasing aggression during exercise, increasing strength, psychological wellbeing	Angoorani [10]; Chandler [12]; Gårevik [18]; Hegazy [23]; Hoff [24]; Hope [25]; Kimergård [30]; Larance [35]; Lundholm [37]; McBride [39]; Pappa [43]; Peters [46]; Skårberg [53,54]; Tallon [56]	15
Muscle oil (synthol)	–	–	Anabolic effect	Skårberg [54]	1
Muscle relaxing drugs^†^	Combating side effects	Klötz [31]	Combating side effects	Klötz [31]	1
Myoblast	NS	Klötz [31]	NS	Klötz [31]	1
Nalbuphine/nubain®	NS	Strauss [55]; Lenehan [36]; McBride [39]	Treating pain from weightlifting injuries, “anti-catabolic”, mental high	Gruber [22]; Hope [25]; Kanayama [27]; Korkia [33]; Lenehan [36]; McBride [39]; Strauss [55]; Wines [58]	8
Naproxen	NS	Strauss [55]	NS	Strauss [55]	1
Non-steroidal anti-inflammatory drugs (NSAIDs) ^†^	–	–	Inflammation, pain, and fever relief	Skårberg [54]	1
Oxycodone	NS	Strauss [55]	NS	Kanayama [27]; Strauss [55]	2
Peptide hormones^†^	–	–	NS	Bilard [11]	1
Phenylbutazone	NS	Strauss [55]	NS	Strauss [55]	1
Phosphodiesterase-5 inhibitors (PDE5i) ^†^	–	–	NS	Hope [25]	1
Piroxicam	NS	Strauss [55]	NS	Strauss [55]	1
Potassium	NS	Strauss [55]	NS	Strauss [55]	1
Potency/testicular increasing drugs^†^	Combating side effects	Klötz [31]	Combating side effects	Klötz [31]	1
Pregnyl®	Improved testosterone production	Peters [46]	Improved testosterone production	Peters [46]	1
Protein powder	Enhancing effects of training, increasing protein synthesis	Perry [45]; Skårberg [53,54]	Enhancing effects of training, increasing protein synthesis	Perry [45]; Skårberg [53,54]	3
Proviron®	Hardiness, improved testosterone production	Peters [46]	Hardiness, improved testosterone production	Peters [46]	1
Recovery drinks^†^	–	–	Endurance booster, recovery aid	Filiault [16]	1
Sedatives^†^	NS	Malone [38]	NS	Malone [38]	1
Sildenafil/viagra®/cialis	Enhanced sexual functioning	Chandler [12]; Kimergård [30]	Enhanced sexual functioning	Chandler [12]; Gårevik [18]; Hope [25]; Kimergård [30]	4
Somatotropine	NS	Klötz [31]	NS	Klötz [31]	1
Spironolactone	Weight loss	Ahlgrim [9]	Weight loss	Ahlgrim [9]	1
Stimulants^†^	NS	Klötz [31]; Kusserow [34]; Malone [38]	Increasing strength, burning fat	Fudala [17]; Klötz [31]; Kusserow [34]; Malone [38]; Rashid [49]; Skårberg [54]	6
Suntan pills^†^	NS	Strauss [55]	NS	Strauss [55]	1
Tamoxifen	Combating side effects	Chandler [12]; Kimergård [30]; Lenehan [36]; McBride [39]	Combating side effects	Chandler [12]; Gruber [22]; Kimergård [30]; Korkia [33]; Lenehan [36]; McBride [39]; Tallon [56]	7
Teroxin (T3)	Preventing weight gain	Peters [46]	Preventing weight gain	Peters [46]	1
Testosterone releasers/boosters^†^	Combating side effects, increasing hormone production	Skårberg [53]	Combating side effects, increasing blood serum levels of testosterone or hormone production	Skårberg [53,54]	2
Thiazides^†^	–	–	NS	McKillop [40]	1
Thiomucase	NS	Wilson-Fearon [57]	Burning fat	Korkia [32,33]; Wilson-Fearon [57]	3
Thyroxine	Burning fat, increasing metabolism	Chandler [12]; Lenehan [36]; Peters [46]; Skårberg [54]; Strauss [55]	Burning fat, increasing metabolism	Chandler [12]; Gruber [22]; Hope [25]; Korkia [32]; Larance [35]; Lenehan [36]; McKillop [40]; Peters [46]; Skårberg [54]; Strauss [55]	10
Tobacco	NS	Malone [38]; Perry [48]; Pope [47]	NS	Malone [38]; Perry [48]; Pope [47]	3
Torsemide	NS	Ahlgrim [9]	NS	Ahlgrim [9]	1
Triacana	–	–	NS	Korkia [33]	1
Yohimbine	NS	Perry [45]	NS	Gruber [22]; Perry [45]	3
Other IPEDs, licit and illicit substances	NS	Kanayama [28]; Klötz [31]; Kusserow [34]; Perry [48]; Skårberg [52]	NS	Gruber [20,21]; Hoff [24]; Hope [25]; Kanayama [26,28]; Klötz [31]; Kusserow [34]; Perry [48]; Skårberg [52,54]; Wines [58]	

IPEDs: Image and performance-enhancing drugs.

NS: Not specified.

^†^Substance class - not specified.

### Lifetime polypharmacy

We also investigated lifetime use of other substances by AAS users. The most popular substances (declared in multiple studies) were: 2,4-dinitrophenol (DNP), alcohol, aminogluthimide, amphetamine/meth, aspirin®, buprenorphine, caffeine, cannabis/cannabinoids, clenbuterol, clomiphene citrate, cocaine, codeine, creatine, ephedra/ephedrine, erythropoietin (EPO), furosemide, gamma hydroxybutyrate (GHB), growth hormone, heroin, human chorionic gonadotropin (hCG), insulin, insulin-like growth factor 1 (IGF-1), ketamine, levodopa, lysergic acid diethylamide (LSD), melanotan, nalbuphine/nubain®, oxycodone, protein powder, sildenafil/viagra®/cialis®, tamoxifen, thiomucase, thyroxine, and yohimbine. Other popular classes of substances presented were analgesics/opioids, antibiotics, anti-catabolics, anti-oestrogens, benzodiazepines, blood pressure regulators, bronchodilators, dietary/nutritional supplements, diuretics, hallucinogens, inhalants, stimulants, and testosterone releasers/boosters.

Of the above substances, the most commonly identified in studies include alcohol, cannabis/cannabinoids, cocaine, growth hormone, human chorionic gonadotropin (hCG), amphetamine/meth, clenbuterol, ephedra/ephedrine, insulin, and thyroxine. Commonly identified classes of substances include analgesics/opioids, dietary/nutritional supplements, diuretics, and anti-oestrogens (see Table 3).

### Groups of non-AAS substances used by AAS users

Our classification of the various substances used by AAS users resulted in 13 main groups: analgesics/non-steroidal anti-inflammatory drugs/opioids, anti-oestrogens, cardiovascular drugs, central nervous system depressants, central nervous system stimulants, cosmetic drugs, dietary/nutritional supplements, diuretics, fat burning/weight loss drugs, muscle/strength-enhancement hormones, non-hormone muscle/strength-enhancement drugs, recreational substances/drugs, and sexual enhancement drugs (see Table 4). These groups of substances are briefly discussed below.

**Table 4:** **Groups of non-AAS substances used by AAS users**

**Group**	**Examples**	**Purpose(s)**
Analgesics/non-steroidal anti-inflammatory drugs/opioids	Acetaminophen, aspirin®, Ben-Gay®, benoxaprofen, buprenorphine, codeine, corticosteroids^†^, heroin, hydrocodone, lidocaine, muscle oil (synthol) and muscle relaxing drugs^†^, nalbuphine/nubain®, naproxen, oxycodone, phenylbutazone, piroxicam	Relieving inflammation, pain, and fever
Anti-oestrogens	Aminogluthimide, aromatase inhibitors^†^, clomiphene/clomid, proviron®, tamoxifen	Improved testosterone production, burning body fat, reducing the effects of AAS on oestrogens, and dealing with gynecomastia
Cardiovascular drugs	Beta-2-agonists^†^, beta-blockers^†^, captopril, carvedilol, digoxin, thiazides^†^	Lowering blood pressure, reducing risk of infarction, and burning body fat
CNS depressants	Alcohol, benzodiazepines^†^, buprenorphine, cannabis/cannabinoids, diazepam, gamma hydroxybutyrate (GHB), heroin, hydrocodone, ketamine, oxycodone	Improving sleep, relaxation, and dealing with side effects of AAS use such as gynecomastia
CNS stimulants	Amyl nitrate, caffeine, cocaine, ephedrine, epinephrine, mephedrone, meth/amphetamine, yohimbine	Alertness, boosting training, burning body fat, increased aggression and strength, and psychological wellbeing
Cosmetic drugs	Anti-acne drugs^†^, esiclene, melanotan I, suntan pills, thiomucase	Curing acne, skin tanning, and enhancing physical appearance
Dietary/nutritional supplements	Calcium, choline and inositol, chromium picolinate, conjugated linoleic acid, creatine, electrolyte solution, glutamine, hydroxocobal amin, piroxicam, potassium, protein powder	For essential nutrients to supplement the diet and combat the risk of illness
Diuretics	Furosemide, hydrochlorothiazide, spironolactone, torsemide	Increasing strength, masking AAS and other doping drugs, burning body fat, and reducing levels of body fluid
Fat burning/weight loss drugs	2,4-dinitrophenol (DNP), anti-oestrogens^†^, beta blockers^†^, bronchodilators^†^, caffeine, chromium picolinate, clenbuterol, cocaine, conjugated linoleic acid, ephedrine, hydrochlorothiazide, insulin^**^, laxatives^††^, liothyronine, melanotan II, meth/amphetamine, spironolactone, teroxin (T3), thiomucase, thyroxine, triacana, yohimbine	Suppression of appetite, increased metabolism, and reduced absorption of body fat
Muscle/strength-enhancement drugs (non-hormone)	Amphetamine/meth, anti-catabolics^†^, glutamine, bronchodilators, chromium picolinate, clenbuterol, creatine, ephedrine, herbal products^††^, hydroxocobal amin (B12), myoblast, muscle oil (synthol), protein powder, recovery drinks^†^	Enhancing the size and structure of muscles as well as boosting strength
Muscle/strength-enhancement hormones^††^	Dehydroepiandrosterone (DHEA), erythropoietin (EPO), genotropine, growth hormone, growth hormone releasing peptide (GHRP), human chorionic gonadotropin (hCG), insulin-like growth factor 1 (IGF-1), insulin, levodopa, mechano growth factor, pregnyl®, prohormones^†^, proviron®, somatotropine	Enhancing the size and structure of muscles as well as boosting strength
Recreational substances/drugs	Alcohol, buprenorphine, cannabis/cannabinoids, cigarettes/tobacco methamphetamine, blood pressure regulators^†^, caffeine, cocaine, ecstasy, hallucinogens^†^, heroin, hydrocodone, ketamine, lysergic acid diethylamide (LSD), sedatives^†^, tetrahydrocannabinol	Enhancing sleep, relaxation, and psychological wellbeing
Sexual enhancement drugs	Anti-oestrogens^†^, human chorionic gonadotropin (hCG), melanotan II, phosphodiesterase-5 inhibitors (PDE5i), sildenafil/cialis®, yohimbine	Dealing with testicular atrophy, improved sexual desire or arousal and boosting erectile functioning

CNS: Central nervous system.

^†^Substance class - not specified.

^††^Some are used for direct muscle enhancing properties and others for counteracting shut-down of endogenous testosterone production.

^**^Skårberg [54].

There may be overlap between classes (e.g. CNS depressants may be used for promoting sleep and for analgesic properties).

Some of the drugs do not have well documented efficacy for their alleged motives for use.

### Analgesics/non-steroidal anti-inflammatory drugs/opioids

These drugs include aspirin®, codeine, and oxycodone. This group of drugs was used for relieving inflammation, pain, and fever emanating from exercise, sports participation or the recreational and occupational activities of AAS users.

### Anti-oestrogens

Anti-oestrogens include aminogluthimide, clomiphene, and tamoxifen. These drugs were used for reducing the oestrogen-like side effects of AAS use such as preventing gynecomastia. They were also used for endurance, improved testosterone production, and burning body fat.

### Cardiovascular drugs

These drugs such as captopril, carvedilol, and digoxin were used for improved functioning of the cardiovascular system such as lowering blood pressure and reducing the risk of myocardial infarction, as well as burning body fat.

### Central nervous system depressants

Examples of depressants are buprenorphine, hydrocodone, and oxycodone. The purposes for which these drugs were used were improved sleep, relaxation, and elevation of mood.

### Central nervous system stimulants

Stimulants including epinephrine, amphetamine/methamphetamine, and yohimbine were used for alertness, boosting training, burning body fat, increased aggression and strength (including sexual), and psychological wellbeing.

### Cosmetic drugs

Cosmetic or aesthetic drugs such as esiclene, melanotan II, and thiomucase were used in order to deal with acne, and for: inflammatory effects on smaller muscles, skin tanning, and a leaner physique thus enhancing physical appearance.

### Dietary/nutritional supplements

These supplements such as calcium, glutamine, and potassium were consumed to provide essential nutrients to supplement the diet and combat the risk of illness.

### Diuretics

Diuretics such as furosemide, hydrochlorothiazide, and spironolactone were used for combating side effects of AAS use such as water retention, together with masking the use of AAS and other doping agents.

### Fat burning/weight loss drugs

These drugs include 2,4-dinitrophenol (DNP), conjugated linoleic acid, and teroxin (T3) and were used for suppression of appetite, increased metabolism, and reduced absorption of body fat as a means to burning body fat and losing weight.

### Muscle/strength-enhancement substances

Two types of muscle/strength-enhancement substances were presented in the literature: hormones and non-hormones. Examples of muscle/strength-enhancement hormones are growth hormones, growth hormone releasing peptide (GHRP), and insulin. Non-hormone muscle/strength-enhancement drugs include clenbuterol used by some in an attempt to enhance the size and structure of muscles, as well as boosting strength.

### Recreational substances/drugs

Recreational substances/drugs such as cannabis/cannabinoids, cocaine, and lysergic acid diethylamide (LSD) were used to alter experiences, elevate mood, and create psychological wellbeing as well as for relaxation.

### Sexual enhancement drugs

These drugs such as phosphodiesterase-5 inhibitors (PDE5i), melanotan II, and sildenafil were used for dealing with testicular atrophy, improved sexual desire or arousal as well as erectile functioning.

In sum, the above groups of substances were used to enhance the effects of AAS, combat the side effects of AAS, and for recreational or relaxation purposes, as well as sexual enhancement. It is important to note that there is overlap between some of the groups. For instance, some central nervous system depressants may be misused for promoting sleep as well as their analgesic properties. Again, some muscle/strength-enhancement hormones are used for direct muscle enhancing properties and others for counteracting shutdown of endogenous testosterone production. Additionally, some of the substances are used for multiple purposes. For instance, melanotan II is used for tanning the skin and also as self–treatment for erectile dysfunction resulting from long-term AAS use. Others may use melanotan II to self-treat specific conditions such as rosacea or fibromyalgia and others may use melanotan for the self-reported weight loss effects due to appetite suppression. It is also important to note that some of the alleged properties or uses are not scientifically well documented such as the use of insulin for burning body fat [54]. Furthermore, the quality, safety, and efficacy of substances obtained from the illicit market cannot be known, with adulteration usually commonplace [2,63].

### Implications for policy and practice

The present study has highlighted various licit and illicit substances used by AAS users. Evidence abounds that some of the substances identified in our study, especially dietary and nutritional supplements, may be contaminated with AAS and other pharmacological elements thus, potentially, playing a role in the decision to initiate AAS use [2,64-67]. Preventive efforts should therefore highlight the potential role licit and illicit substance use, especially dietary and nutritional supplement use, may play in the initiation of AAS use as well as the role AAS use may potentially play in the use of other substances, together with the potential negative consequences individuals who engage in such behavior may encounter.

AAS-associated polypharmacy is dangerous for several reasons. First, it has been associated with violent and criminal behavior as well as various forms of pathology and mortality [68-70]. Second, chemical interactions from AAS-related polypharmacy may have adverse psychophysical effects on individuals engaged in such behavior. Thus, the main and combined effects of the use of these substances must attract the attention of clinicians, policymakers and public health officials. Indeed, physicians may inadvertently administer medication to AAS-using polydrug users thereby triggering unintended adverse chemical interactions that may be harmful to AAS-using patients. Accordingly, gathering correct and comprehensive substance use histories of AAS users is important in the effective pharmacological and psychological treatment of AAS users [67,71] as such information may guide clinicians in the diagnosis and prescription of ‘safe’ drugs during treatment.

Additionally, most AAS users obtain the substances identified in the present study from the illicit market [1,2]. Because many of these substances are controlled or illegal [2], they may be produced in unsterile ‘underground laboratories’ leading to inadvertent and sometimes deliberate incorrect dosing, substitution of ingredients and contamination with additional pharmaceuticals, toxic chemicals and pathogens. Furthermore, some users resort to unsterile injection equipment for the administration of these products, resulting in injecting site injuries as well as bacterial and fungal infection [72] and the potential transmission of blood borne viruses such as hepatitis B/C and HIV [25]. Stakeholders must take our findings into consideration in the development of preventive and therapeutic interventions for AAS users. There is also the need for the strengthening of harm reduction interventions to combat the harmful consequences of AAS-related polydrug use.

### Implications for research

There is the need for further investigations to elucidate better the pathway to AAS-associated polysubstance use. Further studies are also necessary to examine the main and complementary enervating consequences of the use of different dosages of these varied substances, plus their addictive potential and trajectories. Moreover, there is a dearth of knowledge regarding the spread of these substances due to the fact that most of these substances are relatively new. So far most focus has been directed toward AAS in particular. Thus, the use of ancillary and associated substances has mainly escaped the attention of clinicians, public health officials, policymakers, and researchers [2]. There is therefore the need for studies examining the emergence of these substances in the pharmacopoeia of substance users as well as their diffusion into other substance-using populations.

There is the need for the collection and analysis or testing of these substances, to ascertain their content and potential contaminants. Additionally, apart from the Iranian study [10], all studies were conducted in Western countries. Nonmedical AAS use is a global public health problem [4] and researchers are encouraged to extend their investigations to non-Western nations. Finally, investigations of AAS-associated polypharmacy must be a continuous process requiring updates as evidence accumulates.

### Strengths and limitations

As far as we are aware, the present study is the pioneering international systematic review and synthesis of qualitative studies on AAS use and polypharmacy. The inclusion of both peer-reviewed and grey literature, as well as literature published before 1995 and after 2009, also distinguishes this review from a previous review [3]. The present study also has some limitations that ought to be taken into consideration when interpreting our findings. First, due to the nature of the present study, it was not statistically possible to establish ‘gateway’ or causal associations between AAS use and use of the other substances. In addition, we were unable to establish the prevalence of the use of these substances by AAS users. Third, some of the studies included in the present study did not specifically investigate AAS users’ intake of other licit and illicit substances. Although these studies present very useful data in respect of the present study, it is plausible that these studies do not present a comprehensive picture of the variety of substances ingested by AAS users. Similarly, the case reports included in the present study may have been published because they are ‘extraordinary’ and may therefore not be representative of the ‘typical’ AAS user. With the relative paucity of literature in this field [73], the inclusion of these studies is in our view still defendable. Finally, there is the possibility that our exclusion of non-English language literature may have biased our results. It should be noted however that this very common practice in terms of reviews and meta-analyses might not necessarily affect findings [62].

## Conclusions

Our findings corroborate previous suggestions of associations between AAS use and the use of a wide range of other licit and illicit substances. AAS-related polypharmacy has potential serious harmful effects for persons who engage in such behavior, which should be of serious public health concern. Clinicians, policymakers, researchers, and public health workers dealing with AAS users must be educated about these issues. Importantly, efforts must be intensified to combat the debilitating effects of AAS-concomitant polypharmacy. Furthermore, there needs to be ongoing research to investigate trends in AAS use and polypharmacy.
